# Longitudinal associations of active commuting with body mass index

**DOI:** 10.1016/j.ypmed.2016.06.014

**Published:** 2016-09

**Authors:** Oliver Tristan Mytton, Jenna Panter, David Ogilvie

**Affiliations:** MRC Epidemiology Unit, UKCRC Centre for Diet and Activity Research (CEDAR), University of Cambridge School of Clinical Medicine, Box 285, Cambridge Biomedical Campus, Cambridge CB2 0QQ, UK

**Keywords:** Adult, Motor activity, Walking, Transportation, Epidemiology, Bicycling, Obesity, Body mass index, Adiposity

## Abstract

**Objective:**

To investigate the longitudinal associations between active commuting (walking and cycling to work) and body mass index (BMI).

**Method:**

We used self-reported data on height, weight and active commuting from the Commuting and Health in Cambridge study (2009 to 2012; n = 809). We used linear regression to test the associations between: a) maintenance of active commuting over one year and BMI at the end of that year; and b) change in weekly time spent in active commuting and change in BMI over one year.

**Results:**

After adjusting for sociodemographic variables, other physical activity, physical wellbeing and maintenance of walking, those who maintained cycle commuting reported a lower BMI on average at one year follow-up (1.14 kg/m^2^, 95% CI: 0.30 to 1.98, n = 579) than those who never cycled to work. No significant association remained after adjustment for baseline BMI. No significant associations were observed for maintenance of walking. An increase in walking was associated with a reduction in BMI (0.32 kg/m^2^, 95% CI: 0.03 to 0.62, n = 651, after adjustment for co-variates and baseline BMI) only when restricting the analysis to those who did not move. No other significant associations between changes in weekly time spent walking or cycling on the commute and changes in BMI were observed.

**Conclusions:**

This work provides further evidence of the contribution of active commuting, particularly cycling, to preventing weight gain or facilitating weight loss. The findings may be valuable for employees choosing how to commute and engaging employers in the promotion of active travel.

## Highlights

• Maintenance of cycling to work was associated with a lower BMI.
• The association was not significant after adjustment for baseline BMI.
• An increase in time walking to work was associated with a reduction in BMI.

## 1 Introduction

Overweight and obesity are major public health concerns. In the UK, it is estimated that obesity costs the National Health Service £5.1 billion per year, and society as a whole £27 billion per year (Public Health England, 2015a). Regular physical activity helps prevent weight gain and aid weight loss (Bull and the Expert Working Groups, 2010, Donnelly et al., 2009), but it can be difficult for people to achieve this in sufficient quantity (Kohl et al., 2012, Scholes and Mindell, 2013). Walking and cycling can be a feasible way for people to incorporate regular physical activity into their daily lives (Sahlqvist et al., 2013, Sahlqvist et al., 2012, Varney et al., 2014). Consequently the promotion of walking and cycling for transport (active travel) has been proposed as one way of helping to reduce the prevalence of obesity (Centres for Disease Control and Prevention, 2010, Varney et al., 2014). Commuting has been a particular focus (Black, 2008, National Institute for Health and Clinical Excellence, 2012, National Institute for Health and Clinical Excellence, 2008a) because it is regularly undertaken by most adults (58% in England & Wales) (Goodman, 2013).

However, the evidence of an aetiological association between active travel and obesity is relatively weak. Some of this evidence is ecological, either from comparisons between countries or cities (Bassett et al., 2008, Pucher et al., 2010), or from temporal trends at the population level showing a rise in obesity coincident with a fall in active travel (Prentice and Jebb, 1995). Other evidence comes from cross-sectional studies of individual-level associations (Flint and Cummins, 2016, Flint et al., 2014, Laverty et al., 2015, Laverty et al., 2013, Lindström, 2008, Millett et al., 2013). None of these studies provide a strong basis from which to infer causation or derive estimates of effect size (Greenland and Robins, 1994, Hill, 1965). These would be better derived from longitudinal studies, of which we are aware of only one concerning active travel and obesity in adults (Martin et al., 2015).

Much existing research has explored active travel as a composite behaviour, often failing to separate walking and cycling (Flint et al., 2014, Laverty et al., 2015, Lindström, 2008, Martin et al., 2015). It may be important to consider the behaviours separately, because the duration and intensity of each activity can differ (Ainsworth et al., 2011, Collins and Mayer, 2015, Millett et al., 2013, Panter et al., 2013) and they tend to be undertaken by different segments of the population, suggesting that they may be differentially associated with body mass index. Some existing research has also failed to adjust for occupational or recreational activity (Laverty et al., 2013, Lindström, 2008, Martin et al., 2015). The aim of this study was therefore to explore the longitudinal associations of both walking and cycling on the commute with body mass index.

## 2 Methods

### 2.1 Study setting and data collection

The analysis used data from the Commuting and Health in Cambridge study, a longitudinal study of commuters working in Cambridge, UK (n = 1431). A full description has been published elsewhere (Ogilvie et al., 2016, Ogilvie et al., 2010). Participants completed up to four annual questionnaires (2009–2012) which included information on travel behaviour, height, weight, physical activity and sociodemographic characteristics. Ethical approval was granted by the Hertfordshire Research Ethics Committee and the Cambridge Psychology Research Ethics Committee. All participants gave written informed consent.

### 2.2 Inclusion and exclusion criteria

Alongside the original cohort, new participants were recruited during each of the first three years of the study. As only a small number of participants completed three or four waves of the study, we restricted our analysis to those who completed two consecutive waves of the study (n = 854). We further excluded those with missing exposure (n = 1), outcome (n = 19) or covariate data (n = 25), such that we undertook a complete case analysis (n = 809). We defined the baseline assessment for each participant as their first assessment with complete information on exposure. The follow-up assessment was completed one year after their baseline questionnaire.

### 2.3 Exposure measures: walking and cycling

The primary exposures of interest were maintenance of cycling to work and maintenance of walking to work. These were ascertained using a seven-day retrospective travel record that asked individuals to report all modes of travel on the journey to work, so capturing multi-modal commuting (MRC Epidemiology Unit, 2009, Ogilvie et al., 2010, Panter et al., 2011). Cycling or walking on any part of any of the reported journey to work was categorised as cycling or walking to work.

While these exposures were ascertained at baseline for each participant, we chose to restrict our analysis to those who were confirmed to have the same exposure at follow-up (i.e. had comparatively stable behaviour during the period of follow-up), as described previously (Mytton et al., 2015). This was to avoid the potential misclassification of those who changed their behaviour during the period of observation. The reference group consisted of those who reported not cycling to work at both baseline and follow-up. Participants who reported no cycling at one time point and cycling at the other time point were excluded from the maintenance analyses.

Evidence of a dose-response relationship between active commuting and BMI might provide additional support for a causal association (Hill, 1965). Previous research suggests that a relatively large ‘dose’ of physical activity is necessary to prevent weight gain or induce weight loss (Bull and the Expert Working Groups, 2010, Donnelly et al., 2009, Swift et al., 2014). Consequently we categorised participants into one of three groups based on their weekly duration of cycle commuting at baseline and follow-up: 0 min, 1–149 min, and > 150 min. Participants who moved between categories between baseline and follow-up were excluded.

The secondary exposures of interest were changes in weekly time spent cycling, and walking, to work. Change in weekly cycle commuting time was estimated by subtracting the weekly cycle commuting time reported at follow-up from the equivalent value at baseline, and categorised into three groups: any increase, no change, and any decrease. We undertook two sensitivity analyses concerning definition of change. The first used the following categories: large increase; no or small change; and large decrease (defining large changes as > 50 min/week, and no or small change as ≤ 50 min/week) (Panter et al., 2015). The second was restricted to those who had not moved home or work location (see Methods Appendix, ‘Change analyses’, for rationale).

The same process was followed for walking to work.

### 2.4 Outcome measures: body mass index (BMI)

We estimated BMI by dividing self-reported weight by the square of self-reported height (World Health Organisation, 2000). Change in BMI was estimating by subtracting baseline from follow-up values. Extreme values for BMI and change in BMI were identified. Height and weight measures were checked against measurements at other time points, and then either modified if we were confident of the true value (n = 8) or deleted if the true value was unclear (n = 2).

### 2.5 Covariates

We hypothesised that several factors may confound the relationship between active commuting and BMI: age, sex, education, physical wellbeing, distance from home to work and other physical activity, with study year included as a co-variate (see Methods Appendix for rationale). Covariates were assessed by questionnaire at baseline. Dates of birth and of questionnaire completion were used to calculate age. Physical Component Score 8 (PCS-8; a measure of physical wellbeing) was derived from the Medical Outcomes Study Short Form 8 by applying standard weights to responses to the different questions (Ware et al., 2001). Physical activity was assessed using the Recent Physical Activity Questionnaire (Besson et al., 2010), from which information on occupational and recreational activities was used to assign one of four levels of non-commuting physical activity similar to those of the Cambridge Physical Activity Index (Wareham et al., 2002) as described previously (Mytton et al., 2015).

### 2.6 Analysis

We used two complementary approaches to test longitudinal associations. First, we modelled the associations of maintenance of cycling, and walking, to work with BMI at follow-up. This may serve as a better test of a temporal relationship, given that the ascertainment of the exposure preceded that of the outcome (Hill, 1965). Second, we modelled the associations of change in cycling, and in walking, to work with change in BMI. This may serve as a better estimate of the effect attributable to a change in behaviour.

The first set of analyses used linear regression adjusted for the covariates hypothesised to act as confounders (Model A) and for study year. We included maintenance of both cycling and walking to work as explanatory variables so that the model estimates were mutually adjusted, because the two behaviours contribute separately to physical activity energy expenditure. To explore the dose-response relationship we repeated the analysis using our three-way categorical measure for both cycling and walking (0 min, 0–149 min, > 150 min). In addition we adjusted the analysis for baseline BMI (i.e. conditional analysis), which we label ‘Model B’. This conditional analysis addresses a different question to the unconditional analysis, namely whether there is a difference in the *change in BMI* between cyclists and non-cyclists *who have the same initial BMI*. It is the most appropriate approach to test for differences in change between two groups, when there are baseline differences in the outcome of interest between groups (Fitzmaurice, 2001, Twisk and Proper, 2005).

The second set of analyses used linear regression to test the associations between changes in weekly cycling (and walking) commute time and change in BMI, applying the same approach to adjustment for covariates (Model A and Model B) as described above.

A summary of analyses and the research questions each addresses is given in the Methods Appendix (Table A1).

We also tested for effect modification by sex and home-work distance, following the findings of previous research (Flint et al., 2014, Martin et al., 2015) and by weight status (BMI ≤ 25 kg/m^2^ vs BMI > 25 kg/m^2^), as we hypothesised that the effect of physical activity on body weight may vary by BMI·(Barte et al., 2014, Hall et al., 2011).

All analyses were conducted in Stata v13.

## 3 Results

The included participants were predominantly women (69.6%) and educated to at least degree level (69.8%), and slightly more than half reported cycling to work (53.9%). Many of those who walked to work (48.5%) also reported some car commuting (vs 27.3% among those who cycled) (Table 1). The prevalence of obesity and overweight (men: 37.8%; women: 33.2%) was lower than the national average for England (67.1% and 57.2% respectively) (Public Health England, 2015b). There were no major differences between participants included in and excluded from the analysis (Results Appendix, Table A2).

### 3.1 BMI and maintenance of cycling to work

After excluding those who stopped or started cycling (n = 110) or walking (n = 144) between the two time points, 579 participants were included in the maintenance analyses. Those who maintained cycling to work had a significantly lower BMI at follow-up, after adjustment for covariates (Table 2, Model A; change in BMI − 1.14 kg/m^2^, 95% CI − 2.00 to − 0.32), than those who did not cycle to work. Adjustment for maintenance of walking strengthened the observed association, and adjustment for home-work distance attenuated it (Model A, without adjustment for maintenance of walking: − 0.86 kg/m^2^, 95% CI − 1.64 to − 0.08; Model A, without adjustment for home-work distance: − 1.45 kg/m^2^, 95% CI − 2.14 to − 0.75). The effect size on BMI of 1–149 min of cycling to work per week was similar to that of cycling 150 min or more (− 1.28 kg/m^2^, 95% CI − 2.32 to − 0.23 vs. − 1.26 kg/m^2^, 95% CI − 2.26 to − 0.27; n = 493). Additionally adjusting the analysis for baseline BMI markedly attenuated the association such that it was no longer significant (Table 2, Model B).

Under Model A significant interactions were observed between maintenance of cycling to work and home-work distance (p = 0.001) and BMI (p = 0.02), but not sex (p = 0.23). Stratifying by home-work distance, a stronger association with BMI was observed among those living further from work (0–9.99 km: 0.04 kg/m^2^, 95% CI − 0.83 to 0.93, n = 395; 10–19.99 km: − 1.27 kg/m^2^, 95% CI − 3.03 to 0.49, n = 105; ≥ 20 km: − 2.77 kg/m^2^, 95% CI − 4.35 to − 1.19, n = 199). Stratifying by weight status, a stronger association was observed among those who were overweight or obese at baseline (− 1.02 kg/m^2^, 95% CI − 2.08 to 0.02, n = 375; vs. 0.05 kg/m^2^, 95% CI − 0.41 to 0.52, n = 204, for those with a BMI ≤ 25 kg/m^2^).

### 3.2 BMI and maintenance of walking to work

There was no significant association between maintenance of walking to work and BMI (Table 2), despite the observation that adjustment for maintenance of cycling strengthened the association (Model A, without adjustment for maintenance of cycling to work: − 0.36 kg/m^2^, 95% CI − 1.13 to 0.43). All specified interactions were non-significant. There was some evidence of a possible dose-response relationship between walking and BMI (1–149 min: − 0.51 kg/m^2^, 95% CI − 1.68 to 0.65; > 150 min: − 0.95 kg/m^2^, 95% CI − 2.36 to 0.47; n = 542), although the differences were not significant.

### 3.3 Change in BMI and changes in weekly time spent cycling or walking to work

There were no significant associations between either change in weekly cycle commute time or change in weekly walking commute time and change in BMI, in our primary analysis (Table 3). Interaction terms for sex, home-work distance and BMI were not significant. When restricting the analysis (n = 651) to those who did not move home or work, a significant association between increase in walking and reduction in BMI was observed (Results Appendix, Table A3). The associations between large increases/decreases and change in BMI were non-significant for both walking and cycling (Results Appendix, Table A4).

## 4 Discussion

### 4.1 Principal findings

We found that maintenance of cycling to work was associated with a lower BMI at one-year follow-up, after adjustment for covariates. This association was stronger for those who had a longer distance to commute or who were overweight or obese at baseline, but there was no evidence of a ‘dose-response’ effect. However the conditional analysis (adjusting for baseline BMI) was not significant. We found that increasing walking was associated with a reduction in BMI, but only when we restricted our analysis to those who had not moved home or work. While other associations for walking were non-significant, the *pattern* of results for walking was consistent with the findings of past research that has observed associations between walking to work and BMI.

### 4.2 Strengths and limitations

The strengths of this study lie in the use of complementary longitudinal analyses and the separation of active commuting into walking and cycling. Although the exposure was self-reported, we have previously shown good agreement between self-reported and objective estimates of time spent in active commuting using this measure (Panter et al., 2014). Moreover, the use of a detailed commuter travel record has enabled us to identify walked or cycled undertaken as part of a longer journey completed by car or public transport. This accurate classification of travel behaviour has allowed us to partially compensate for the smaller size of our study compared to other studies with coarser measures of travel (Flint et al., 2014, Martin et al., 2015), and although our sample size may appear modest, we had sufficient power to detect some positive associations.

The outcome was self-reported and is prone to systematic biases in reporting, in that heavier individuals tend to under-report their body weight (Crawley and Portides, 1995, Park et al., 2011). As heavier participants were less likely to report active commuting in our study, this reporting bias may have attenuated the observed associations. Conversely, because the study was designed to investigate the relationships between commuting and health, it is possible that some responses may have been affected by a social desirability bias whereby those who travelled by active or ‘healthy’ means were more likely to under-report their body weight. Such a bias would have strengthened the observed relationship.

Although our analyses were adjusted for the complementary commuting activity (walking or cycling) and other forms of physical activity, we have not adjusted for other aspects of behaviour, which were not captured but are associated with BMI (e.g. diet or sleep). Nor have we adjusted for car driving, which was not well captured (time or distance not recorded) (McCormack and Virk, 2014, Swanson and McCormack, 2012). Although, it is unclear to what extent the effect of car driving on BMI is due to an absence of active travel. Cambridge has a high prevalence of cycling compared to the national average (29% cycle vs 3% based on ‘usual mode’ of commuting reported in the 2011 Census) (Office for National Statistics, 2013) and we also note that the study population was relatively affluent, educated and predominantly white-collar (Goodman et al., 2012, Humphreys et al., 2013, Panter et al., 2015). Consequently the findings may not be generalizable to other groups.

### 4.3 Comparison with other studies

Our findings broadly corroborate and build on the existing literature, providing further evidence of inverse associations between active travel and BMI·(Flint and Cummins, 2016, Flint et al., 2014, Laverty et al., 2015, Laverty et al., 2013, Lindström, 2008, Martin et al., 2015, Millett et al., 2013).

Taken together our findings for walking appear weaker than those for cycling, but are consistent with the literature. Other studies have found smaller effects for walking than for cycling (Flint and Cummins, 2016), or only observed associations in those who undertake more walk commuting (Laverty et al., 2013, Millett et al., 2013). Others defined walkers as those who used walking as the ‘main mode’ of travel (Martin et al., 2015), in contrast we defined walkers as those who undertook any walking on their commute and consequently included many people who used other travel modes. The relatively low average quantity of walking to work (median 90 min/week) or relatively high car use among walkers may also have contributed to the non-significant findings.

Ours estimates of effect size are consistent with estimates from other studies. For example, our results for change in walking are comparable to a previous effect estimate of 0.3 kg/m^2^ for commuters changing from the car to active travel, while our results for maintenance of cycling are comparable to cross-sectional estimates of a difference of 0.7 to 1.1 kg/m^2^ between active commuters and those using private motor vehicles (Flint and Cummins, 2016, Flint et al., 2014, Martin et al., 2015).

### 4.4 Interpretation and implications

We conducted two sets of complementary longitudinal analyses. We had hypothesised that the maintenance analyses might provide a test of a temporal relationship. Although the exposure was ascertained prior to the outcome, the pattern of results (with a null association after conditioning on baseline BMI) could be explained by baseline BMI determining the likelihood of cycling (i.e. acting as a confounder). Such an explanation would undermine an argument about the biological plausibility of a causal effect of active commuting on BMI. However the findings are also consistent with the explanation that cycling to work prior to baseline contributed to differences in baseline BMI. From our analyses we cannot distinguish between these alternative explanations, and consequently one should be cautious about drawing unequivocal causal inference from the findings.

Only one set of change analyses was significant that for non-movers whose walking increased. While the sample size for the change analyses was larger than for the maintenance analyses, they may have had less power to detect an association. First, the exposure of participants may have been misclassified if other factors (e.g. annual leave, weather or variable work commitments) produced an *apparent* change in travel behaviour between the two time points, biasing the association towards the null. Our experience from the maintenance analyses suggested that removing misclassified participants produced stronger associations. Second, there is a lag between changes in physical activity and the full change in BMI·(Hall et al., 2011). Given that the change in active commuting could have happened at any time between baseline and follow-up, the study design is unlikely to have permitted observation of the full effect of changes in active commuting on BMI. Third, other changes may have co-occurred with the change in active travel that might influence BMI in either direction and could not readily be accounted for. We note that excluding movers from the analysis (who might be subject to other life changes that could influence BMI) tended to strengthen the observed associations. One should therefore be cautious of over-interpreting the null results from the change analyses.

Taken together our findings provide some evidence that active commuting, particularly cycling, may contribute to preventing weight gain or facilitating weight loss. The effect estimates may appear comparatively small from the individual perspective, in that 1.2 kg/m^2^ equates to a difference of 3 kg for a person 1.6 m or 5 ft 3 in. tall. However at a population level such differences are important, given an average weight gain of 10 kg in the US during the thirty years when obesity prevalence among adults has risen from around 10% to over 35% (Hall et al., 2011, US Department of Health and Human Services and National Insti). This suggests that increasing active commuting could be an important component of a strategy for reducing or preventing obesity. This is an important message not only for individuals choosing how to commute or governments prioritising investment in transport infrastructure, but also employers who can influence the social, economic and environmental determinants of active travel among their employees (Black, 2008, Dalton et al., 2013, National Institute for Health and Clinical Excellence, 2012, National Institute for Health and Clinical Excellence, 2008b, Sallis et al., 2012).

We observed a significant interaction between maintenance of cycling and home-work distance, with a stronger association between maintenance of cycle commuting and BMI among those who lived 20 km or more from work, mirroring previous findings (Martin et al., 2015). This could reflect those living further from work being at greater risk of obesity (those who lived 20 km or more from work had a mean BMI of 25.2, compared to 23.7 for those who lived within 10 km) perhaps because of reduced time for activities that prevent weight gain (e.g. healthy eating or sleep) (García-Fernández et al., 2014, Wu et al., 2014). Equally it may reflect residual confounding by age, SES (e.g. high living costs in Cambridge) (Goodman et al., 2012), or other covariates (Swanson and McCormack, 2012). In keeping with this finding, we also observed a stronger absolute effect estimate among those who were overweight at baseline. Taken together these findings suggest a particularly valuable role for active commuting among populations who are more liable to be obese.

### 4.5 Unanswered questions and future research

Considerable uncertainty remains concerning the dose, frequency and intensity of active travel necessary to prevent weight gain or stimulate weight loss. Future research should seek to reduce this uncertainty. Obesity is a proxy for adiposity. Future studies should use objective measures of adiposity to increase the precision of the outcome measures (Wong et al., 2003) and to shed mechanistic insight into the relationship between active travel and cardio-metabolic disease by testing associations with specific types of body fat linked to disease (Fox et al., 2007, Preis et al., 2010). There is a suggestion in our study of differential effects for those who live further from work and those who are obese. Future work should seek to explore the effect of active travel on adiposity in different groups, particularly those who have a greater BMI or are more predisposed to obesity, including car commuters.

## 5 Conclusions

Our work provides some evidence of the potential contribution of active commuting to preventing weight gain or reducing BMI among adults of working age. Our findings may be important for individuals choosing how to commute, employers seeking to improve the health of their workforce, and governments seeking to adopt policies to prevent obesity.

## Conflict of interest

The authors declare there is no conflict of interest.

## Transparency document

Transparency document. Image 1

## Figures and Tables

**Table 1:** Baseline characteristics of participants included in the analyses (n = 809).

	Cycling to work		Walking to work	
	None (n = 373)	Some (n = 436)	None (n = 597)	Some (n = 204)
	N (%)	N (%)	N (%)	N (%)
*Gender*				
Female	289 (51.3)	274 (48.7)	197 (80.1)	49 (19.9)
Male	84 (34.2)	162 (65.9)	408 (72.5)	155 (27.5)

*Age*				
Median (years)	44.1 (34.8–52.9)	42.9 (33.1–51.5)	43.3 (34.0–52.0)	43.4 (42.7–52.8)
16–29 years	42 (39.6)	64 (60.4)	73 (68.9)	33 (31.1)
30–39 years	106 (47.5)	117 (52.5)	170 (76.2)	53 (23.8)
40–49 years	95 (44.6)	118 (55.4)	165 (77.5)	48 (22.5)
50–59 years	94 (46.5)	108 (53.5)	151 (74.8)	51 (25.2)
≥ 60 years	36 (55.4)	29 (44.6)	46 (70.8)	19 (29.2)

*Highest educational qualification*				
Less than degree	142 (58.2)	102 (41.8)	183 (75.0)	61 (25.0)
Degree or higher	231 (40.9)	334 (59.1)	422 (74.7)	143 (25.3)

*Weight status*				
Underweight/normal weight	217 (41.0)	312 (59.0)	400 (75.6)	129 (24.4)
Overweight	105 (50.2)	104 (49.8)	152 (72.7)	57 (27.3)
Obese	51 (71.8)	20 (28.2)	53 (74.7)	18 (25.4)

*Body Mass Index (kg/m^2^)*				
Median (IQR)	24.4 (21.5–27.3)	23.3 (21.4–25.4)	23.7 (21.5–26.3)	23.6 (21.3–26.4)

*PCS-8 score*				
Median (IQR)	55.2 (51.1–58.0)	55.7 (52.5–58.0)	55.4 (51.7–58.0)	55.4 (51.4–58.1)

*Home-work distance*				
0.01–9.99 km	120 (25.3)	355 (74.7)	361 (76.8)	109 (23.2)
10–19.99 km	71 (61.2)	45 (38.8)	89 (76.7)	27 (23.3)
≥ 20 km	182 (83.5)	36 (16.5)	147 (68.4.0)	68 (31.6)

*Physical activity index*				
Inactive	9 (37.5)	15 (62.5)	19 (79.2)	5 (20.8)
Moderately inactive	115 (50.9)	109 (49.1)	168 (74.3)	58 (25.7)
Moderately active	113 (46.5)	132 (53.5)	178 (73.3)	65 (26.7)
Active	136 (43.0)	180 (57.0)	240 (76.9.0)	76 (24.1)

*Weekly time cycling to work*				
Median (IQR) (min)	0 (0–0)	150 (90–200)	90 (0–180)	0 (0 − 30)

*Weekly time walking to work*				
Median (IQR) (min)	0 (0–90)	0 (0–0)	0 (0–0)	100 (60–180)

*Use of other modes for commuting*				
Car	245 (65.7)	119 (27.3)	265 (43.8)	99 (48.5)
Public transport	115 (30.8)	43 (9.9)	64 (10.6)	94 (46.1)

*Changed behaviour*				
Started walking/cycling to work	43 (11.5)	0 (0)	76 (12.6)	0 (0)
Stopped walking/cycling to work	0 (0)	67 (15.4)	0 (0)	68 (33.3)

*Time frame*				
2009–10	313 (47.5)	346 (52.5)	486 (73.8)	173 (26.3)
2010–11	15 (39.5)	23 (60.5)	23 (60.5)	15 (39.5)
2011–12	45 (40.2)	67 (59.8)	96 (85.7)	16 (14.3)

IQR = Interquartile range; PCS-8 = Physical Component Summary score derived from the Short Form 8 Questionnaire, theoretical score range is 9.1 to 69.0, with a mean of 50 in the US adult population; unless otherwise stated characteristics are measured at baseline; changed behaviour describes the number of individuals who started or stopped active travel between baseline and follow-up (e.g. cycle to work at baseline and not cycling to work at follow-up); use of other modes, includes any use of the stated mode to commute to or from work, including for part of the journey, in the past seven days; car use includes the use taxi); multimodal commuting indicates that the behaviour was combined with either car use or public transport; Study undertaken in Cambridge, UK (2009–12).

**Table 2:** Associations of maintenance of cycling to work and maintenance of walking to work with BMI (n = 579).

		Unadjusted	Model A	Model B
		Coefficient (95% CI)	Coefficient (95% CI)	Coefficient (95% CI)
Cycling to work	None (reference)			
	Some	**− 1.25 (− 1.83, − 0.67)**	**− 1.14 (− 1.98, − 0.30)**	− 0.12 (− 0.42, 0.17)
Walking to work	None (reference)			
	Some	− 0.19 (− 0.99, 0.62)	− 0.80 (− 1.63, 0.04)	− 0.18 (− 0.48, 0.11)
Gender	Male (reference)			
	Female	**− 0.71 (− 1.41, − 0.00)**	**− 0.80 (− 1.49, − 0.11)**	− 0.02 (− 0.26, 0.22)
Age	16–29 years (reference)			
	30–39 years	**1.62 (0.49, 2.77)**	**1.28 (0.17, 2.39)**	− 0.17 (− 0.56, 0.22)
	40–49 years	**2.23 (1.09, 3.37)**	**1.77 (0.66, 2.88)**	0.06 (− 0.32, 0.45)
	50–59 years	**2.65 (1.51, 3.80)**	**2.29 (1.17, 3.40)**	− 0.16 (− 0.56, 0.22)
	≥ 60 years	**2.94 (1.48, 4.41)**	**2.09 (0.64, 3.53)**	0.07 (− 0.44, 0.57)
Degree	No (reference)			
	Yes	**− 1.03 (− 1.75, − 0.31)**	**− 0.78 (− 1.51, − 0.04)**	0.01 (− 0.24, 0.27)
Home-work distance	0.01–9.99 km (reference)			
	10–19.99 km	0.73 (− 0.19, 1.65)	0.06 (− 0.90, 1.03)	− 0.14 (− 0.48, 0.19)
	≥ 20 km	**1.41 (0.65, 2.16)**	0.64 (− 0.26, 1.53)	− 0.08 (− 0.40, 0.23)
Physical wellbeing (PCS-8)		**− 0.08 (− 0.13, − 0.02)**	− 0.05 (− 0.10, 0.00)	− 0.02 (− 0.04, − 0.00)
Physical activity	Inactive (reference)			
	Moderately inactive	**− 3.67 (− 5.82, − 1.52)**	**− 3.44 (− 5.54, − 1.34)**	0.19 (− 0.55, 0.93)
	Moderately active	**− 4.72 (− 6.86, − 2.57)**	**− 4.33 (− 6.44, − 2.22)**	0.22 (− 0.53, 0.96)
	Active	**− 4.30 (− 6.42, − 2.17)**	**− 4.07 (− 6.16, − 1.99)**	0.34 (− 0.39, 1.08)
Study year	2009–10 (reference)			
	2010–11	− 0.67 (− 2.23, 0.88)	− 0.11 (− 1.60, 1.37)	− 0.09 (− 0.60, 0.43)
	2011–2	− 0.27 (− 1.24, 0.69)	− 0.43 (− 1.38, 0.52)	− 0.06 (− 0.39, 0.27)
Baseline BMI		**0.94 (0.91, 0.96)**	–	**0.94 (0.91, 0.97)**

Linear regression coefficients shown; - not included; CI = confidence interval; PCS-8 = Physical Component Summary score derived from the Short Form 8 questionnaire; physical activity is categorised using a modified form of the Cambridge Physical Activity Index; study year refers to the time period when data were collected; bold indicates significant results (p < 0.05); Model A is adjusted for gender, age, education, home-to-work distance, physical wellbeing, physical activity and study year; Model B is adjusted for adjusted for gender, age, education, home-to-work distance, physical wellbeing, physical activity, study year and BMI at baseline; Study undertaken in Cambridge, UK (2009–12).

**Table 3:** Associations of changes in weekly cycle commuting time and weekly walking commuting time with change in BMI (n = 809).

		Unadjusted	Model A	Model B
		Co-efficient (95% CI)	Co-efficient (95% CI)	Co-efficient (95% CI)
Cycling to work	No change (reference)			
	Increase in weekly time (n = 182)	0.14 (− 0.09, 0.37)	0.09 (− 0.15, 0.34)	0.06 (− 0.18, 0.31)
	Decrease in weekly time (n = 224)	0.16 (− 0.06, 0.38)	0.15 (− 0.08, 0.39)	0.14 (− 0.10, 0.37)
Walking to work	No change (reference)			
	Increase in weekly time (n = 139)	− 0.20 (− 0.45, 0.05)	− 0.20 (− 0.45, 0.04)	− 0.23 (− 0.48, 0.02)
	Decrease in weekly time (n = 126)	0.25 (− 0.01, 0.51)	0.24 (− 0.02, 0.50)	0.25 (− 0.01, 0.50)

Linear regression coefficients shown; CI = confidence interval; Model A is adjusted for age, education, sex, study year, home-work distance, Physical Component Summary score derived from the Short Form 8 questionnaire, physical activity categorised using a modified form of the Cambridge Physical Activity Index; Model B is adjusted for age, education, sex, study year, home-work distance, Physical Component Summary score derived from the Short Form 8 questionnaire, physical activity categorised using a modified form of the Cambridge Physical Activity Index and baseline BMI; Study undertaken in Cambridge, UK (2009–12).

## Appendix A Supplementary data

Supplementary material 1. Image 2

Supplementary material 2. Image 3
