# Supplementary material for: Longitudinal associations of active commuting with body mass index
Source: Prev Med. 2016 Sep;90:1–7. doi: 10.1016/j.ypmed.2016.06.014 (PMC5023394; doi:10.1016/j.ypmed.2016.06.014)
Supplement: Supplementary file 2 — Supplementary material 1. [file mmc2.docx]

**Methods Appendix**

**Rationale for inclusion of co-variates**

Age and sex: BMI varies with age and sex.(Craig and Mindell, 2014). Both are also determinants of active travel.(Goodman et al., 2012; Guell et al., 2012; Panter et al., 2013, 2011)

Socio-economic status: Obesity is patterned by SES.(Craig and Mindell, 2014) In previous analyses SES has been shown to be associated with active travel.(Goodman et al., 2012; Panter et al., 2013, 2011) In the Commuting and Health in Cambridge study we had a choice of two measures of SES (area-level deprivation from home postcode, and education status). We chose education status because it is an individual measure (rather than area-level measure) and in our data, it was strongly associated with BMI for both men and women. The further addition of an area-level measure of deprivation (quintile of index of multiple deprivation) did not materially alter the model findings.

Physical well-being: We hypothesised that poor physical health could restrict ability to walk or cycle to work. Univariable analysis in our dataset supported this rationale. We also hypothesised that poor physical health would restrict ability to be active in other areas of life (beyond that captured by recreational physical activity). Reduced physical activity in other areas of life could also affect BMI.(Donnelly et al., 2009; Warburton et al., 2010)

Other physical activity: Physical activity is associated with BMI.(Donnelly et al., 2009; Warburton et al., 2010) It is commonly suggested that individuals who travel actively may also be active in other areas of their life,(Flint and Cummins, 2016) and thus it is important to adjust for other forms of physical activity (although on univariate analysis in our dataset we did not find an association between active travel and physical activity, assessed using our modified Cambridge Physical Activity Index).

Home-work distance: Home-work distance was associated with active commuting in our study sample (Goodman et al., 2012; Panter et al., 2012, 2011) and associated with other factors such as SES (the price of housing in Cambridge is high, forcing some people to live out of Cambridge and commute into the city). Commute duration is associated with reduced well-being.(Office for National Statistics, 2014; Roberts et al., 2011) Lengthy commuting can also reduce time available for other health-promoting activities (e.g. sleep, healthy eating, leisure-time physical activity) and might therefore conceivably be associated with obesity by those other pathways. Home-work distance was also strongly associated with BMI in our dataset.

Study year: Study year was also included as a covariate as we drew participants from three different years of entry to the cohort.

Change analyses

We applied the same covariates for the two approaches (association between maintenance or walking or cycling and BMI at follow-up; association between change in walking or cycling and change in BMI) to testing longitudinal associations. For the second approach (association between change in walking or cycling and change in BMI), we considered adjusting for time varying covariates (i.e. other variables that may have changed and might confound the relationship), but such variables were either unavailable (e.g. change in diet or change in sleep), liable to be on the causal pathway (e.g. change in recreational physical activity, which might arise as a result of change in commuting physical activity leading to a change in well-being and change in other non-commuting physical activity), or of uncertain association with BMI (e.g. change in home location, which might be associated with a set of other changes in e.g. sleep, time pressure or stress that might influence BMI in either direction). Adjustment for variables on the causal pathway (i.e. mediators) would be inappropriate.(Bauman et al., 2002) Adjustment for variables whose association with BMI is uncertain or mixed (i.e. could be a positive or negative confounder) is unlikely to aid comprehension. A change in home (or work) location may be one such variable. Whether it acts as a positive or negative confounder may depend on other contextual factors. Consequently we chose to undertake a sensitivity analysis in which the change analyses restricted to those who had not moved home or work.

**Figure A1: Directed acyclic graph showing the hypothesised relationship between active commuting and body mass index**

**
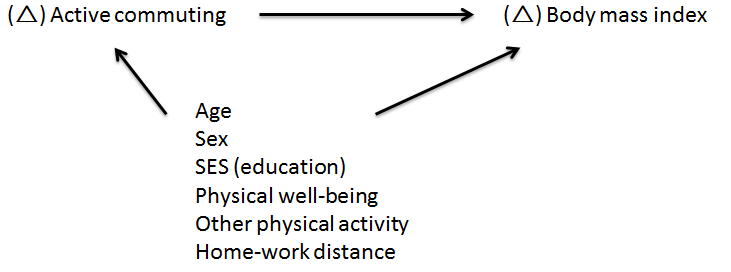
**

**Table A1: Summary of analyses and research questions considering cycle commuting***

| **Exposure** | **Categorisation** | **Outcome** | **Adjustment** | **Research question** |
| --- | --- | --- | --- | --- |
| Maintenance of cycling | None vs some | BMI at one-year follow-up | Model A | What is the difference in body mass index at one-year follow-up between those who maintain commuting by bicycle during the year of follow-up and those who maintain not doing so, after adjustment for covariates? |
| Maintenance of cycling | None vs some | BMI at one-year follow-up | Model B (conditional) | What is the difference in *change* in body mass index at one-year follow-up between those who maintain commuting by bicycle during the year of follow-up and those who maintain not doing so, after adjustment for covariates *assuming the same baseline body mass index*? |
| Maintenance of cycling | 0 minutes per week; 1-149 minutes per week; >150 minutes per week | BMI at one-year follow-up | Model A | What is the difference in body mass index at one-year follow-up between those who maintain commuting: a) by bicycle for at least 150 minutes per week; b) by bicycle for 1-149 minutes per week; and c) not commuting by bicycle, after adjustment for covariates?  This serves as a test for a dose-response relationship. |
| Maintenance of cycling | 0 minutes per week; 1-149 minutes per week; >150 minutes per week | BMI at one-year follow-up | Model B | What is the difference in body mass index at one-year follow-up between those who maintain commuting: a) by bicycle for at least 150 minutes per week; b) by bicycle for 1-149 minutes per week; and c) not commuting by bicycle, after adjustment for covariates *assuming the same baseline body mass index*?  This serves as a test for a dose-response relationship. |
| Change in time per week | No change, increase, decrease | Change in BMI (follow-up BMI minus baseline BMI) | Model A | What is the difference in change in body mass index between those whose cycle commuting time: a) increased; b) decreased; and c) did not change, after adjusting for co-variates? |
| Change in time per week | No change, increase, decrease | Change in BMI (follow-up BMI minus baseline BMI) | Model B (conditional) | What is the difference in change in body mass index between those whose cycle commuting time: a) increased; b) decreased; and c) did not change, after adjusting for co-variates *assuming the same* baseline BMI? |
| Change in cycling time per week | No or small change, large increase (> 50 minutes per week), large decrease (>50 minutes per week) | Change in BMI (follow-up BMI minus baseline BMI) | Model A | What is the difference in change in body mass index between those whose cycle commuting time: a) increased by 50 minutes or more per week; b) decreased by 50 minutes or more per week; and c) did not change or changed by less than 50 minutes per week, after adjusting for co-variates? |
| Change in cycling time per week | No or small change, large increase (> 50 minutes per week), large decrease (>50 minutes per week) | Change in BMI (follow-up BMI minus baseline BMI) | Model B (conditional) | What is the difference in change in body mass index between those whose cycle commuting time: a) increased by 50 minutes or more per week; b) decreased by 50 minutes or more per week; and c) did not change or changed by less than 50 minutes per week, after adjusting for co-variates *assuming the same* baseline BMI? |
| Change in time per week (excluding movers) | No change, increase, decrease | Change in BMI (follow-up BMI minus baseline BMI) | Model A | What is the difference in change in body mass index between those whose cycle commuting time: a) increased; b) decreased; and c) did not change, after adjusting for co-variates among those whose active commuting time changed for reasons other than changing work or home location?  This may be a better test of the effect of a change *in active commuting* on BMI as it only considers those whose activity pattern changed whilst continuing to commute between the same home and work locations. |
| Change in time per week (excluding movers) | No change, increase, decrease | Change in BMI (follow-up BMI minus baseline BMI) | Model B (conditional) | What is the difference in change in body mass index between those whose cycle commuting time: a) increased; b) decreased; and c) did not change, after adjusting for co-variates and *assuming the same* baseline BMI, among those whose active commuting time changed for reasons other than changing work or home location?  This may be a better test of the effect of a change *in active commuting* on BMI as it only considers those whose activity pattern changed whilst continuing to commute between the same home and work locations. |

Model A co-variates: age, sex, education, physical well-being, other physical activity and home-work distance; Model B co-variates: age, sex, education, physical well-being, other physical activity, home-work distance and baseline BMI; * an analogous set of questions apply to walking.
