# Supplementary material for: Longitudinal associations of active commuting with body mass index
Source: Prev Med. 2016 Sep;90:1–7. doi: 10.1016/j.ypmed.2016.06.014 (PMC5023394; doi:10.1016/j.ypmed.2016.06.014)
Supplement: Supplementary file 3 — Supplementary material 2. [file mmc3.docx]

**Results Appendix**

**Table A2: Characteristics of participants included and excluded from the analyses**

|  | **Included (n=809)** | **Excluded** |
| --- | --- | --- |
|  | **N (%)** | **N (%)** |
| **Gender** |  |  |
| Female | 563 (69.6) | 409 (69.1) |
| Male | 246 (30.4) | 183 (30.9) |
| **Age** |  |  |
| Median (IQR) | 43.3 (33.7-52.2) | 38.4 (31.0-48.1) [n=599] |
| 16-29 years | 106 (13.1) | 133 (22.2) |
| 30-39 years | 223 (27.6) | 193 (32.3) |
| 40-49 years | 213 (26.3) | 150 (25.0) |
| 50-59 years | 202 (25.0) | 87 (14.5) |
| ≥60 years | 65 (8.0) | 36 (6.0) |
| **Highest educational qualification** |  |  |
| Less than degree | 244 (30.2) | 183 (31.2) |
| Bachelor or higher | 565 (69.8) | 403 (68.8) |
| **Weight status** |  |  |
| Normal or underweight | 529 (65.4) | 346 (60.0) |
| Overweight | 209 (25.8) | 164 (28.4) |
| Obese | 71 (8.8) | 67 (11.6) |
| **BMI (kg/m^2^)** |  |  |
| Median (IQR) | 23.7 (21.5-26.3) | 24.1 (21.8-27.1) |
| **PCS-8 score** |  |  |
| Median (IQR) | 55.5 (51.5-58.0) | 55.1 (51.5-57.7) [n=594] |
| **Home to work distance** |  |  |
| Median (IQR) | 8.0 (4.8-22.5) | 8.0 (3.2-20.9) [n=600] |
| 0-9.99 km | 475 (58.7) | 344 (57.3) |
| 10-19.99 km | 116 (14.3) | 105 (17.5) |
| ≥20 km | 218 (27.0) | 151 (25.2) |
| **Physical activity index** |  |  |
| Inactive | 24 (3.0) | 20 (3.3) |
| Moderately inactive | 226 (27.9) | 159 (26.6) |
| Moderately active | 243 (30.0) | 202 (33.8) |
| Active | 316 (39.1) | 217 (36.3) |
| **Weekly time cycling to work (minutes)** |  |  |
| Median (IQR) | 50 (0-150) | 0 (0-150) [n=602] |
| **Weekly time walking to work (minutes)** |  |  |
| Median (IQR) | 0 (0-10) | 0 (0-50) [n=597] |

IQR=Interquartile range; PCS-8 = Physical Component Summary score derived from the Short Form 8 Questionnaire; MCS-8 = Mental Component Summary score derived from the Short Form 8 Questionnaire; deprivation quintile is based on national quintiles of deprivation ranked using the Index of Multiple Deprivation 2010 score for the Lower Super Output Area (assigned based on postcode or residence).

**Table A3: Associations of changes in weekly cycle commuting time and weekly walking commuting time with change in BMI, restricted to those who did not move home or work (n=651)**

|  |  | **Unadjusted** | **Model A** | **Model B** |
| --- | --- | --- | --- | --- |
|  |  | Co-efficient  (95% CI) | Co-efficient  (95% CI) | Co-efficient  (95% CI) |
| **Cycling to work** | No change (reference) |  |  |  |
|  | Increase in weekly time (n=141) | 0.21 (-0.06, 0.48) | 0.19 (-0.09, 0.48) | 0.15 (-0.13, 0.43) |
|  | Decrease in weekly time (n=169) | 0.21 (-0.04, 0.46) | 0.25 (-0.02, 0.53) | 0.23 (-0.05, 0.50) |
|  |  |  |  |  |
| **Walking to work** | No change (reference) |  |  |  |
|  | Increase in weekly time (n=105) | **-0.29 (-0.58, -0.00)** | **-0.29 (-0.59, -0.00)** | **-0.32 (-0.62,-0.03)** |
|  | Decrease in weekly time (n=101) | **0.30 (0.01, 0.59)** | 0.29 (-0.01, 0.59) | 0.29 (-0.01, 0.59) |

Linear regression coefficients shown; CI=confidence interval; bold indicates significant results (p<0.05); Model A is adjusted for age, education, sex, study year, home-work distance, Physical Component Summary score derived from the Short Form 8 questionnaire, physical activity categorised using a modified form of the Cambridge Physical Activity Index; Model B is adjusted for age, education, sex, study year, home-work distance, Physical Component Summary score derived from the Short Form 8 questionnaire, physical activity categorised using a modified form of the Cambridge Physical Activity Index and baseline BMI; Study undertaken in Cambridge, UK (2009-12).

**Table A4: Associations of large changes (≥ 50 minutes per week) in weekly cycling and walking commuting time with change in BMI (n=809)**

|  |  | **Unadjusted** | **Model A** | **Model B** |
| --- | --- | --- | --- | --- |
|  |  | Co-efficient  (95% CI) | Co-efficient  (95% CI) | Co-efficient  (95% CI) |
| **Cycling to work** | No change (reference) |  |  |  |
|  | Increase in weekly time (n=106) | 0.16 (-0.12, 0.44) | 0.12 (-0.16, 0.41) | 0.12 (-0.17, 0.40) |
|  | Decrease in weekly time (n=149) | 0.21 (-0.03, 0.45) | 0.20 (-0.05, 0.45) | 0.20 (-0.05, 0.45) |
|  |  |  |  |  |
| **Walking to work** | No change (reference) |  |  |  |
|  | Increase in weekly time (n=76) | -0.23 (-0.55, 0.08) | -0.23 (-0.55, 0.09) | -0.24 (-0.56, 0.07) |
|  | Decrease in weekly time (n=80) | 0.29 (-0.02, 0.59) | 0.25 (-0.06, 0.57) | 0.27 (-0.04, 0.58) |

Linear regression coefficients shown; CI=confidence interval; Model A is adjusted for age, education, sex, study year, home-work distance, Physical Component Summary score derived from the Short Form 8 questionnaire, physical activity categorised using a modified form of the Cambridge Physical Activity Index; Model B is adjusted for age, education, sex, study year, home-work distance, Physical Component Summary score derived from the Short Form 8 questionnaire, physical activity categorised using a modified form of the Cambridge Physical Activity Index and baseline BMI; Study undertaken in Cambridge, UK (2009-12).
